# TREK-1 Channel Expression in Smooth Muscle as a Target for Regulating Murine Intestinal Contractility: Therapeutic Implications for Motility Disorders

**DOI:** 10.3389/fphys.2018.00157

**Published:** 2018-03-06

**Authors:** Ruolin Ma, Mohsen Seifi, Maria Papanikolaou, James F. Brown, Jerome D. Swinny, Anthony Lewis

**Affiliations:** School of Pharmacy and Biomedical Sciences, Institute of Biological and Biomedical Sciences, University of Portsmouth, Portsmouth, United Kingdom

**Keywords:** TREK-1, smooth muscle, ileum, colon, K_2P_ channels, contractility, motility

## Abstract

Gastrointestinal (GI) motility disorders such as irritable bowel syndrome (IBS) can occur when coordinated smooth muscle contractility is disrupted. Potassium (K^+^) channels regulate GI smooth muscle tone and are key to GI tract relaxation, but their molecular and functional phenotypes are poorly described. Here we define the expression and functional roles of mechano-gated K_2P_ channels in mouse ileum and colon. Expression and distribution of the K_2P_ channel family were investigated using quantitative RT-PCR (qPCR), immunohistochemistry and confocal microscopy. The contribution of mechano-gated K_2P_ channels to mouse intestinal muscle tension was studied pharmacologically using organ bath. Multiple K_2P_ gene transcripts were detected in mouse ileum and colon whole tissue preparations. Immunohistochemistry confirmed TREK-1 expression was smooth muscle specific in both ileum and colon, whereas TREK-2 and TRAAK channels were detected in enteric neurons but not smooth muscle. In organ bath, mechano-gated K_2P_ channel activators (Riluzole, BL-1249, flufenamic acid, and cinnamyl 1-3,4-dihydroxy-alpha-cyanocinnamate) induced relaxation of KCl and CCh pre-contracted ileum and colon tissues and reduced the amplitude of spontaneous contractions. These data reveal the specific expression of mechano-gated K_2P_ channels in mouse ileum and colon tissues and highlight TREK-1, a smooth muscle specific K_2P_ channel in GI tract, as a potential therapeutic target for combating motility pathologies arising from hyper-contractility.

## Introduction

Gastrointestinal motility is a highly complex physiological process involving the coordinated contractions of the *tunica muscularis*, a layer of the outer wall of the alimentary canal formed of two sheets of circularly and longitudinally orientated smooth muscle cells. Disruption to the natural rhythm of intestinal contractions can lead to common hypo- or hyper-motility pathologies such as irritable bowel syndrome (IBS) (Beyder and Farrugia, 2012).

The process of smooth muscle cell excitation-contraction coupling occurs predominantly via calcium influx through plasma membrane voltage-dependent calcium channels (VDCC). The activity of VDCC is intimately linked to cell membrane potential (Sanders, 2008; Jepps et al., 2009), which is set primarily by potassium-selective (K^+^) channels (Koh et al., 2012). K^+^ channels are formed from multimers of subunits containing either a single (K_1P_) or tandem (K_2P_) pore domain structure. A multitude of voltage-dependent and voltage-independent K_1P_ channels have been shown to be expressed, both molecularly and functionally, in smooth muscles of the gastrointestinal tract (for review see Koh et al., 2012).

In contrast, our understanding of the expression of the voltage-independent K_2P_ channel subfamily in gastrointestinal tissues is limited. K_2P_ channels contribute to the generic background potassium conductance in many cell types (Feliciangeli et al., 2015) and can be regulated by a plethora of endogenous and environmental cues including changes to oxygen, temperature, and pH levels or membrane tension (Maingret et al., 1999; Lewis et al., 2001; Kang et al., 2005). A functional role in myogenic regulation has, however been proposed (for review see Sanders and Koh, 2006). First described as a stretch-dependent potassium (SDK) conductance in mouse colonic smooth muscle cells, it bears all the biophysical hallmarks of a mechano-gated TREK-like channel including sensitivity to mechanical stretch, insensitivity to tetraethylammonium (TEA) and 4-aminopyridine (4-AP) and regulation by nitric oxide (Koh and Sanders, 2001; Koh et al., 2001; Park et al., 2005). However, direct pharmacological validation of TREK K_2P_ channels in gastrointestinal smooth muscle is currently lacking.

Here we use a combined molecular, protein and pharmacological approach to study the expression of K_2P_ channels in both ileum and colon of mouse, with focus on the specific distribution and functional role of the mechano-gated subfamily members TREK-1, TREK-2, and TRAAK.

## Methods

### Experimental animals

All procedures involving experimental animals were approved by the Animal Welfare and Ethics Review Committee of the University of Portsmouth and were performed in accordance with regulations issued by the Home Office of the United Kingdom under the Animals (Scientific Procedures) Act, 1986. All animals were housed in standard plastic cages, on sawdust bedding in an air-conditioned room at 22 ± 1°C under lighting controls with 12 h light and dark cycles. Standard mouse chow and tap water were provided *ad libitum*. Adult (2–3 months) wild type (WT) C57BL/6J mice (Charles River Laboratories, Margate, UK) of either sex were used throughout the study.

### Quantitative RT-PCR

Quantitative reverse transcription-PCR (qRT-PCR) was used to define the expression profiles of K_2P_ transcripts from ileum and colon tissue sections, as previously described (Papanikolaou et al., 2017). Custom primers (Primerdesign Ltd, Chandlers Ford, UK) were designed to target the 14 identified mouse K_2P_ genes (mice lack the KCNK17 gene encoding for TALK-2), as detailed in Supplementary Table 1. Mice (*n* = 3) were killed by cervical dislocation and the segments of the ileum and colon manually dissected and snap frozen in liquid nitrogen. Frozen tissue was homogenized in RNase-free and sterile conditions and RNA extracted using an RNeasy Midi Kit (Qiagen, Manchester, UK) following the manufacturer's protocol. RNA was then reverse transcribed into single-stranded cDNA using the RT^2^ First Strand Kit (Qiagen, Manchester, UK). Quantity and quality of both RNA and cDNA was assessed spectrophotometrically. Quantitative RT-PCR (qPCR) was conducted in triplicate from generated cDNA libraries using SYBR green qPCR Mastermix (Qiagen, Manchester, UK) on Roche Lightcycler 96 (Roche, Burgess Hill, UK). Relative gene expression was determined using the 2^−Δ*Ct*^ method vs. GAPDH which was identified as the most appropriate housekeeping gene using the Normfinder algorithm (Andersen et al., 2004) and the standard deviation (*SD*) method (Mane et al., 2008).

### Tissue preparation for immunohistochemistry

Mice were anesthetized with isoflurane and pentobarbitone (1.25 mg kg^−1^ bodyweight, i.p.). Animals were transcardially perfused using a fixative containing 1% paraformaldehyde and 15% v/v saturated picric acid in 0.1 M phosphate buffer (pH 7.4), as previously described (Seifi et al., 2014). After perfusion, the ileum and colon were surgically removed and post-fixed in the same fixative overnight at 4°C, before washing in 0.1 M phosphate buffer to clear residual fixative. Whole-mount preparations of the longitudinal muscle-myenteric plexus (MP) and circular muscle-submucosal plexus were obtained using a dissecting microscope and fine forceps, and stored in 0.1 M phosphate buffer containing 0.05% sodium azide at 4°C until use.

### Immunohistochemistry

Specificity of primary antibodies has been confirmed previously, as detailed in Supplementary Table 2, and therefore was not re-examined here. Non-specific binding of secondary antibodies was blocked by incubating the tissue with 20% normal horse serum, diluted in Tris buffer saline containing 0.3% Triton X-100 (TBS-Tx) for 2 h at room temperature. Tissue sections were then incubated with cocktails of primary antibodies (listed in Supplementary Table 2), diluted in TBS-Tx and 20% normal horse serum, overnight at 4°C on a rotating platform. Tissues were then washed with TBS-Tx three times and incubated in a mixture of appropriate conjugated secondary antibodies (see Supplementary Table 2) for 2 h at room temperature. Tissues were washed three times in TBS-Tx and mounted on glass slides in Mowiol mounting medium (Polysciences, Hirschberg an der Bergstrasse, Germany) under a cover slip. Secondary antibody specificity was assessed by omitting the primary antibodies in the incubation sequence. To confirm the absence of cross reactivity between IgGs in double and triple immunolabeling experiments, some sections were processed using only a single primary antibody with the full complement of secondary antibodies. Immunohistochemical labeling was determined by confocal microscopy based on 3–4 sections for each antibody from *n* = 3 animals.

### Image capture and analysis

Sections were examined with a confocal laser-scanning microscope (LSM710; Zeiss) using either a Plan Apochromatic 40x DIC oil objective (NA 1.3; pixel size 0.29 μm) or a Plan Apochromatic 63x oil objective (NA 1.4; pixel size 0.13 μm). Z-stacks were used for routine evaluation of labeling. All images shown represent a single optical section. Images were acquired using sequential acquisition of different channels to avoid crossover between fluorophores, with the pinholes adjusted to one airy unit for all channels. Identical settings were used to image negative controls. Images were processed using Zen2009 Light Edition software (Zeiss, Cambridge, UK) and exported into Adobe Photoshop CC 2015.5. Only brightness and contrast were adjusted for the whole frame, and no part of a frame was enhanced or modified in any way.

### Organ bath pharmacology

Mice were killed by cervical dislocation and the distal ileum and colon removed and immediately transferred to Krebs' solution containing the following (in mM): 118 NaCl, 4.7 KCl, 1.2 KH_2_PO_4_, 25 NaHCO_3_, 11 glucose, 1.2 MgSO_4_, 2.5 CaCl_2_. The intraluminal contents were gently removed by flushing with the Kreb's solution. Segments of ileum and colon (~2–3 cm) from the same animal were longitudinally mounted in parallel on aerators and suspended by cotton thread from force transducers (Harvard, UK) and both submerged in 10 ml organ bath chambers filled with pre-warmed Kreb's solution (32°C for pre-contracted studies/37°C to measure spontaneous activity), and gassed with carbogen (95% O_2_ and 5% CO_2_). Changes in tension at the transducer were processed through an amplifier (Harvard, Cambridge, UK) and recorded by a dedicated data acquisition system (Power LabChart7, AD Instruments, Oxford, UK). The apparatus was calibrated and tissues placed under 1 g of resting tension and allowed to equilibrate for at least 20 min. Viability of ileum and colon tissues was assessed in parallel by exposure to the parasympathomimetic carbachol (CCh) at concentrations ranging from 10^−9^ to 10^−4^ M. Responses to K_2P_ channel modulators were performed after exposure to submaximal concentrations of CCh or 60 mM KCl and quantified by assessing maximum changes in steady state tension. In separate experiments, spontaneous contractile activity was quantified. Tissues were prepared as described above. After stable baselines were established, changes in the force and frequency induced by K_2P_ modulators were recorded and quantified over 5 min periods pre- and post-drug application.

### Data and statistical analysis

Statistical analyses and graphical evaluations were performed with Prism 7.0 (GraphPad, Inc., La Jolla, CA, USA). Figure legends provide *n* values and specific statistical tests performed. For all analysis, *n* values represent animal numbers. All data are presented as the arithmetic mean ± SEM unless stated otherwise. Changes in tension were normalized to steady-state pre-contracted tension and are reported as percent, and was used to control for natural biological variation in contraction amplitudes of the tissues. Curve fitting for determination of IC_50_ values was performed using the non-linear regression normalized response function. Statistical comparisons were made using either Student's *t*-test (paired or unpaired where appropriate) or two-way ANOVA (not repeated measures), followed by the Bonferroni *post-hoc* test. A *p* < 0.05 was considered statistically significant.

### Drugs

All drugs and chemical were purchased from Sigma Aldrich (Gillingham, UK) unless otherwise stated. Riluzole, BL-1249, Flufenamic acid (FFA), and Cinnamyl-3,4-dihydroxy-α-cyanocinnamate (CDC) were dissolved in DMSO. DMSO final bath concentrations did not exceed 0.1% and had no significant effect on measured tension of the amplitude or frequency of spontaneous muscle contractions (data not shown).

## Results

### K_2P_ channel genes show differential expression in whole ileum and colon tissues of mouse

**Figure 1:**
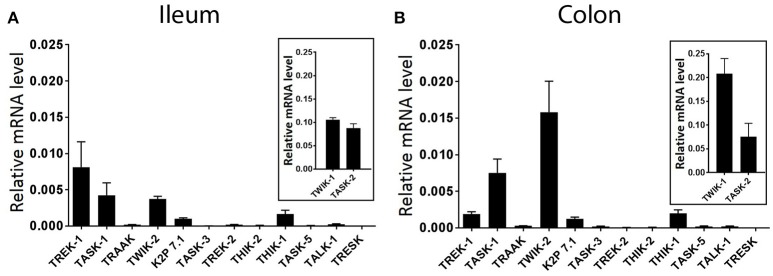
Differential expression of K_2P_ subtype transcripts in the mouse ileum and colon. Quantitative, real-time PCR performed on lysates of acutely isolated ileum **(A)** and colon **(B)** from wild type mice. Data are expressed as relative mRNA levels (2^−Δ*Ct*^) compared to the housekeeping gene GAPDH (mean ± SEM, *n* = 3). TWIK-1, (KCNK1), and TASK-2 (KCNK5) displayed the highest relative expression levels in both ileum and colon (insets). Rank order of detectable expression was as follows, in ileum; TWIK-1 (KCNK1) > TASK-2 (KCNK5) > TREK-1 (KCNK2) > TASK-1 (KCNK3) > TWIK-2 (KCNK6) > THIK-1 (KCNK13) > K_2P_ 7.1 (KCNK7), and in colon; TWIK-1 (KCNK1) > TASK-2 (KCNK5) > TWIK-2 (KCNK6) > TASK-1 (KCNK3) > TREK-1 (KCNK2) > THIK-1 (KCNK13) > K_2P_ 7.1 (KCNK7).

The goal of the current study was to assess the specific distribution and functional contribution of mechano-gated K_2P_ channels in ileum and colon of the mouse. However, a comprehensive assessment of K_2P_ channel expression is currently lacking. We therefore first quantified the expression levels of all known mouse K_2P_ channel genes by real-time PCR (qPCR) analysis, normalized against the housekeeping gene GAPDH by the comparative 2^−Δ*Ct*^ method. qPCR analysis revealed variable expression of multiple K_2P_ channel mRNA transcripts in whole tissue of wild type mouse ileum and colon (Figure 1). Of the 14 K_2P_ channel subunit genes analyzed, both TWIK-1 (KCNK1) and TASK-2 (KCNK5) displayed the highest relative transcript level expression in both whole ileum and colon, an order of magnitude higher than the next most abundant transcripts TREK-1 (KCNK2, ileum) and TWIK-2 (KCNK6, colon). In both tissues, gene transcripts encoding for TRAAK (KCNK4), TASK-3 (KCNK9), TREK-2 (KCNK10), THIK-2 (KCNK12), TASK-5 (KCNK15), and TALK-1 (KCNK16) appeared barely detectable when normalized to housekeeping gene GAPDH and may be suggestive of cell specific expression patterns or broad tissue expression at low levels. Transcript encoding for TRESK (KCNK18) was not detected in either ileum or colon as concluded from a lack of a measurable Ct value after 40 cycles of PCR.

### TWIK-1 and TASK-2 channel proteins display broad distribution patterns in mouse ileum and colon

**Figure 2:**
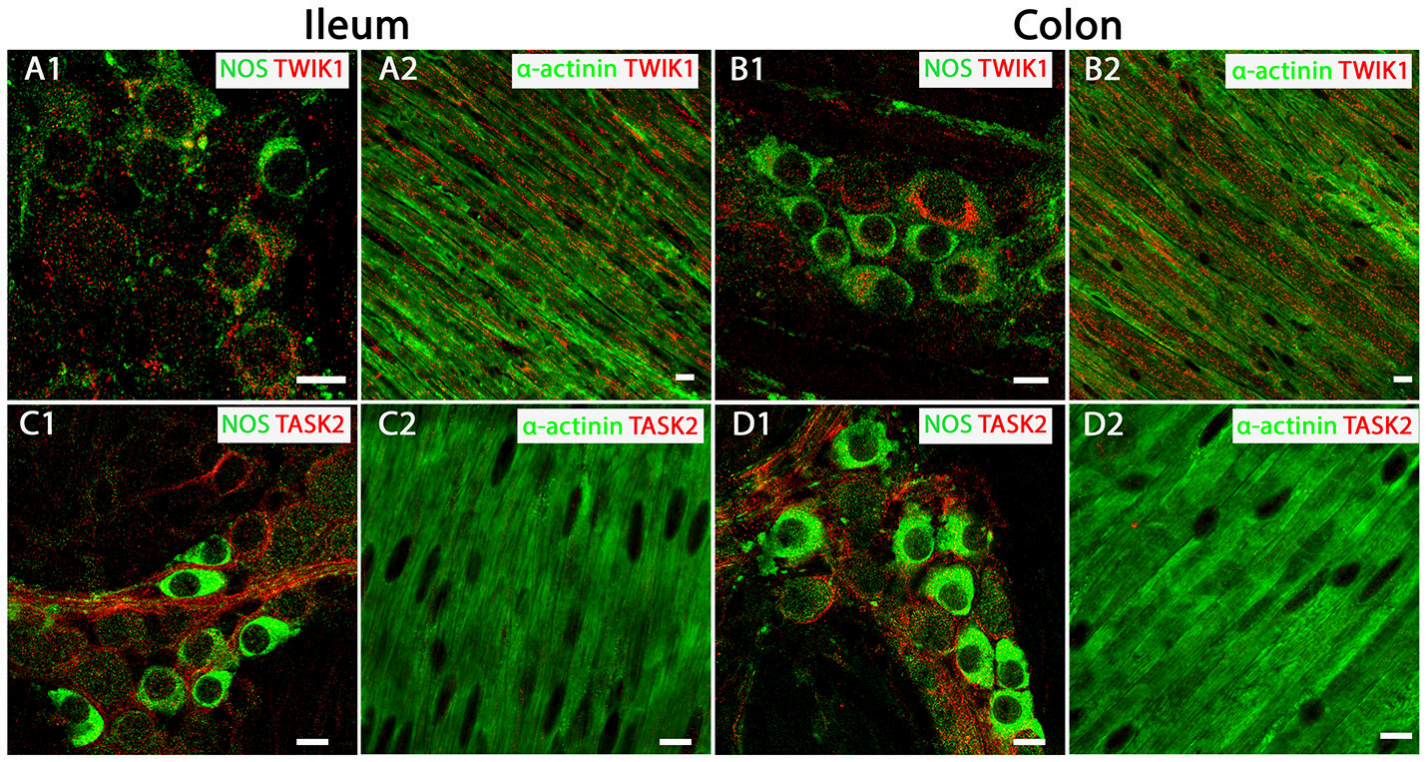
TWIK-1 and TASK-2 channels are distributed in myenteric plexus inhibitory neurons and longitudinal smooth muscle layers of the mouse ileum and colon. Panels show overlays of cell specific markers (green) and individual K_2P_ channel markers (red) throughout. **(A1,B1)** show overlays for NOS immunoreactive myenteric plexus neurons and the TWIK-1 channel in ileum and colon respectively. TWIK-1 labeling was cytoplasmic and can be observed in both NOS-positive and NOS-negative enteric neurons. Within the longitudinal smooth muscle layer, TWIK-1 channels were distributed in α-actinin-immunopositive smooth muscles in both ileum **(A2)** and colon **(B2)**. **(C1,D1)** show overlays for NOS immunoreactive myenteric plexus neurons and the TASK-2 channel in ileum and colon respectively. TASK-2 displayed cell membrane labeling of NOS-negative cell bodies and axons. Immunoreactivity for the TASK-2 channel was not detectable in α-actinin immunopositive longitudinal smooth muscle cells in either mouse ileum **(C2)** or colon **(D2)**. Scale bars represent 10 μm.

Given their abundant transcript levels we determined the precise cellular location TWIK-1 and TASK-2 channels within the mouse intestine, using immunohistochemistry and confocal microscopy. Expression of TWIK-1 was detected on plasma membranes and in cytoplasmic compartments of NOS-immuno-positive MP neurons of both mouse ileum and colon (Figures 2A1,B1). Immunoreactivity for TWIK-1 was also observed in the longitudinal smooth muscle (LM) cells in both ileum and colon, as demonstrated by co-localization with the smooth muscle cell marker α-actinin (Figures 2A2,B2). Immunoreactivity for the TASK-2 channel was significantly enriched in the cell membranes of NOS-immunopositive MP neurons (Figures 2C1,D1), but was not detectable in LM cells of either mouse ileum or colon (Figures 2C2,D2). These data indicate that TWIK-1 and TASK-2 channels are widely distributed across neuronal and smooth muscle tissues and correlates with the abundant transcript expression observed in both ileum and colon. However, a current lack of specific pharmacological tools for TWIK-1 and TASK-2 prevents further detailed functional studies.

### TREK-1 protein shows smooth muscle specific expression in mouse GI tract

Our qPCR data indicate that TREK subfamily channel gene transcripts (TREK-1, TREK-2, and TRAAK) are present in both mouse ileum and colon, however, their levels may be suggestive of cell specific expression patterns. Therefore, we utilized K_2P_ channel specific antibody immunolabeling to characterize the distribution of TREK family channels in muscle and nervous tissue of the mouse GI tract. For consistency with organ bath studies we have focused on expression profiles in LM layers and MP neurons, but duplicate experiments were performed in circular smooth muscle layers and neighboring submucosal plexus neurons, as illustrated in Supplementary Figure 1.

**Figure 3:**
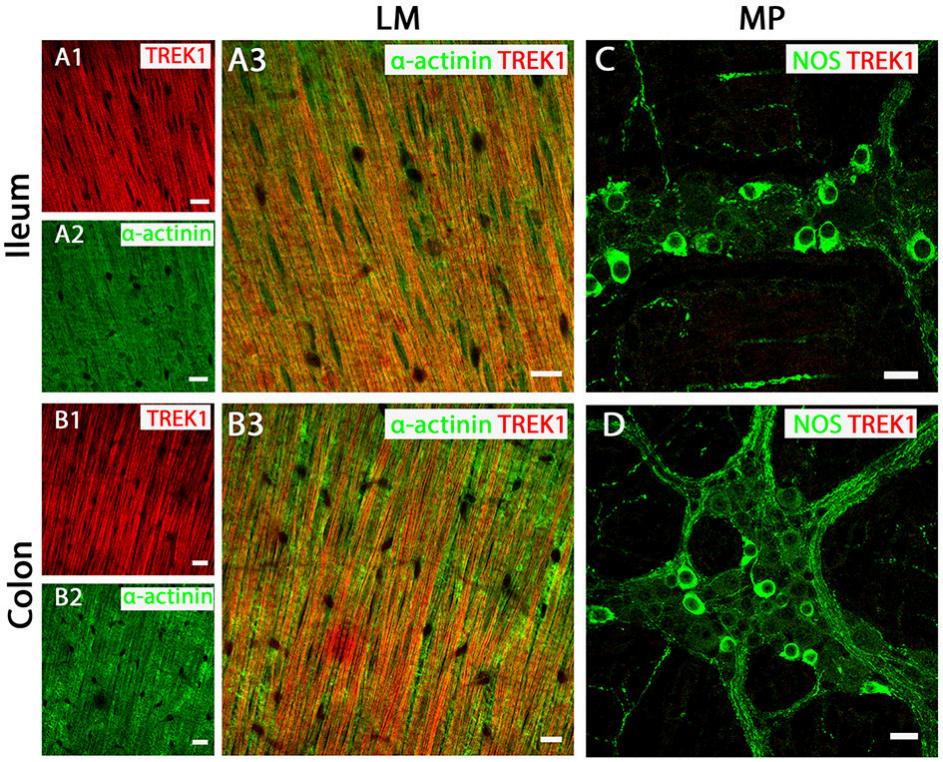
TREK-1 channel are expressed in longitudinal smooth muscle (LM) tissue of both mouse ileum and colon. **(A3,C)** (ileum) and **(B3,D)** (colon) show overlays of cell specific markers (*green*) and the TREK-1 channel marker (*red*). For clarity, (**A1**,**A2)** (ileum) and (**B1,B2)** (colon) show individual images from corresponding positive merges in smooth muscle. Within the longitudinal smooth muscle layer, TREK-1 channels were distributed in α-actinin-immunopositive smooth muscles in both ileum **(A3)** and colon **(B3)**. **(C,D)** show overlays for NOS immunoreactive myenteric plexus (MP) neurons and the TREK-1 channel in ileum and colon respectively. A signal corresponding to the TREK1 channel subunit was not detected in neuronal tissue of either ileum or colon. Scale bars represent 10 μm.

Immunoreactivity for the TREK-1 channel indicated it was widely distributed across longitudinal (Figures 3A,B) and circular (Supplementary Figure 1) smooth muscle tissue in both ileum and colon, as visualized by overlapping immunoreactivity with the smooth muscle cell marker α-actinin. In contrast, TREK-1 immunoreactivity was not detected in NOS-immunopositive MP neurons and submucosal plexus neurons (Figures 3C,D and Supplementary Figure 1).

### TREK-2 and TRAAK proteins are not expressed in GI tract smooth muscle

**Figure 4:**
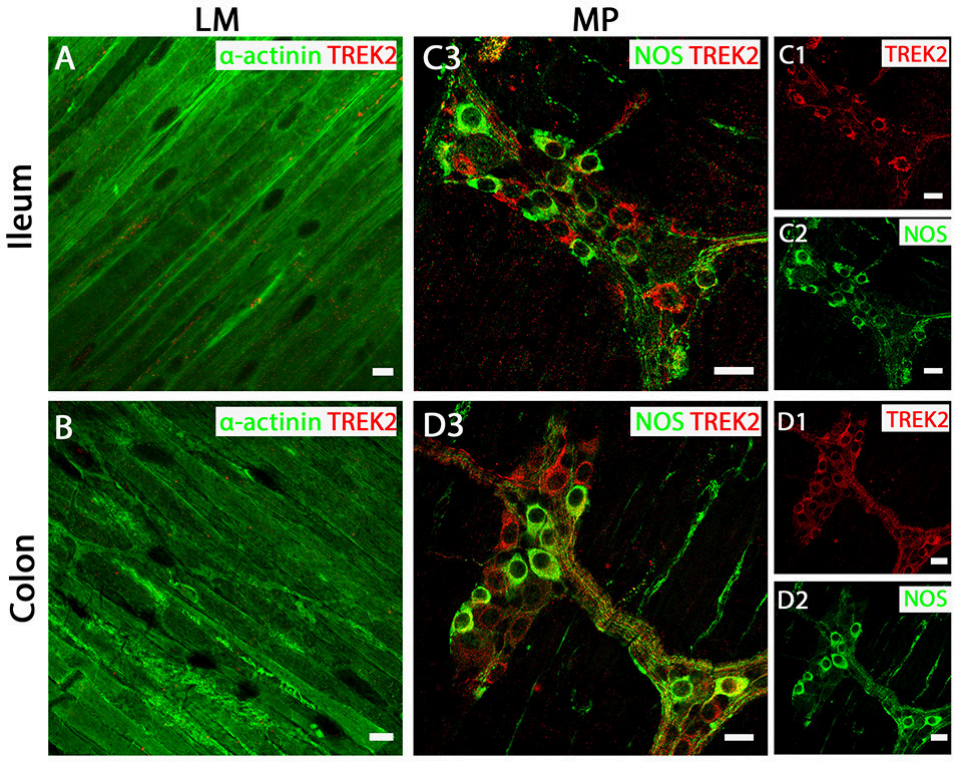
TREK-2 channels are expressed in myenteric plexus (MP) neurons of both mouse ileum and colon. **(A,C3)** (ileum) and (**B,D3)** (colon) show overlays of cell specific markers (*green*) and the TREK-2 channel marker (*red*). For clarity, **(C1**,**C2)** (ileum) and (**D1,D2)** (colon) show individual images from corresponding positive merges in enteric neurons. Within the longitudinal smooth muscle (LM) layer, the TREK-2 channel signal was weak and barely detectable in α-actinin immunopositive smooth muscle cells in both ileum **(A)** and colon **(B)**. **(C3,D3)** show overlays for NOS immunoreactive myenteric plexus neurons and the TREK-2 channel in ileum and colon respectively. TREK-2 channels appear to be distributed in both NOS-positive and NOS-negative enteric neurons. Scale bars represent 10 μm **(A,B)** and 20 μm **(C1–C3,D1–D3)**.

**Figure 5:**
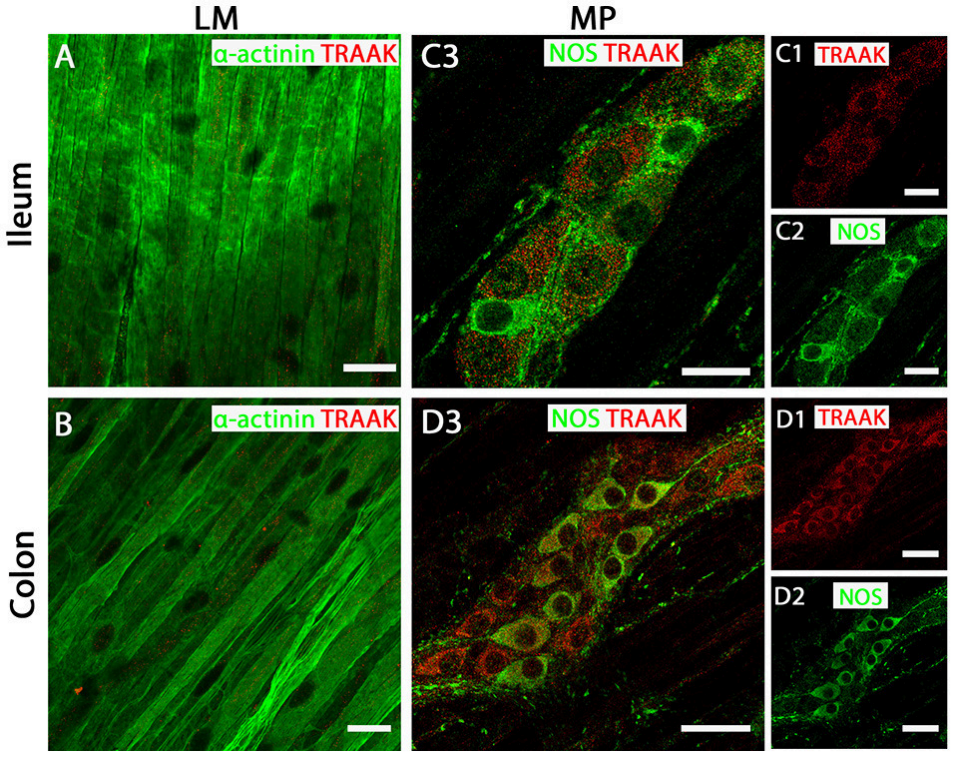
TRAAK channels are expressed in myenteric plexus (MP) neurons of both mouse ileum and colon. **(A**,**C3)** (ileum) and **(B,D3)** (colon) show overlays of cell specific markers (*green*) and the TRAAK channel marker (*red*). For clarity, **(C1**,**C2)** (ileum) and **(D1**,**D2)** (colon) show individual images from corresponding positive merges in enteric neurons. Within the longitudinal smooth muscle (LM) layer, the TRAAK channel signal was weak and barely detectable in α-actinin immunopositive smooth muscle cells in both ileum **(A)** and colon **(B)**. **(C3,D3)** show overlays for NOS immunoreactive myenteric plexus neurons and the TRAAK channel in ileum and colon respectively. TRAAK channels appear to be distributed in the cytoplasm of both NOS-positive and NOS-negative enteric neurons. Scale bars represent 20 μm **(A,B,C1–C3)** and 40 μm **(D1–D3)**.

In both mouse ileum and colon, we were unable to detect immunoreactivity for the TREK-2 channel in LM cell layers (Figures 4A,B). In contrast, strong TREK-2 labeling was observed within NOS-immunopositive MP neurons (Figures 4C,D). Moreover, TREK-2 was also detected in a number of non-NOS enteric neurons and cholinergic neurons (Figures 4C,D) as visualized by co-labeling with the acetylcholine synthesizing enzyme, choline acetyltransferase (ChAT) and the calcium binding protein, calretinin (Supplementary Figure 2). Similar to TREK-2, we could find no evidence for TRAAK expression in LM cells in either mouse ileum or colon (Figures 5A,B). The pattern of TRAAK channel labeling observed in the MP neurons of ileum and colon was analogous to that observed for TREK-2, displaying a wide distribution across both NOS-immunopositive and NOS-immunonegative cell types of MPs (Figures 5C,D).

Collectively, these data reveal the tissue specific distribution of TREK subfamily K_2P_ channels in mouse ileum and colon, with TREK-1 confined to smooth muscle and TREK-2 and TRAAK channels displaying neuronal expression with no detectable expression in smooth muscle.

### TREK family activators regulate the force but not frequency of spontaneous contractions

Previous reports have revealed an important stretch-activated K^+^ conductance SDK in colonic smooth muscle cells that bares all the hallmarks of a background TREK-like channel current (Koh and Sanders, 2001; Koh et al., 2001; Park et al., 2005). However, direct pharmacological evidence for the specific involvement of mechano-gated K_2P_ subfamily channels in ileum and colon smooth muscle contractility is pauce.

**Figure 6:**
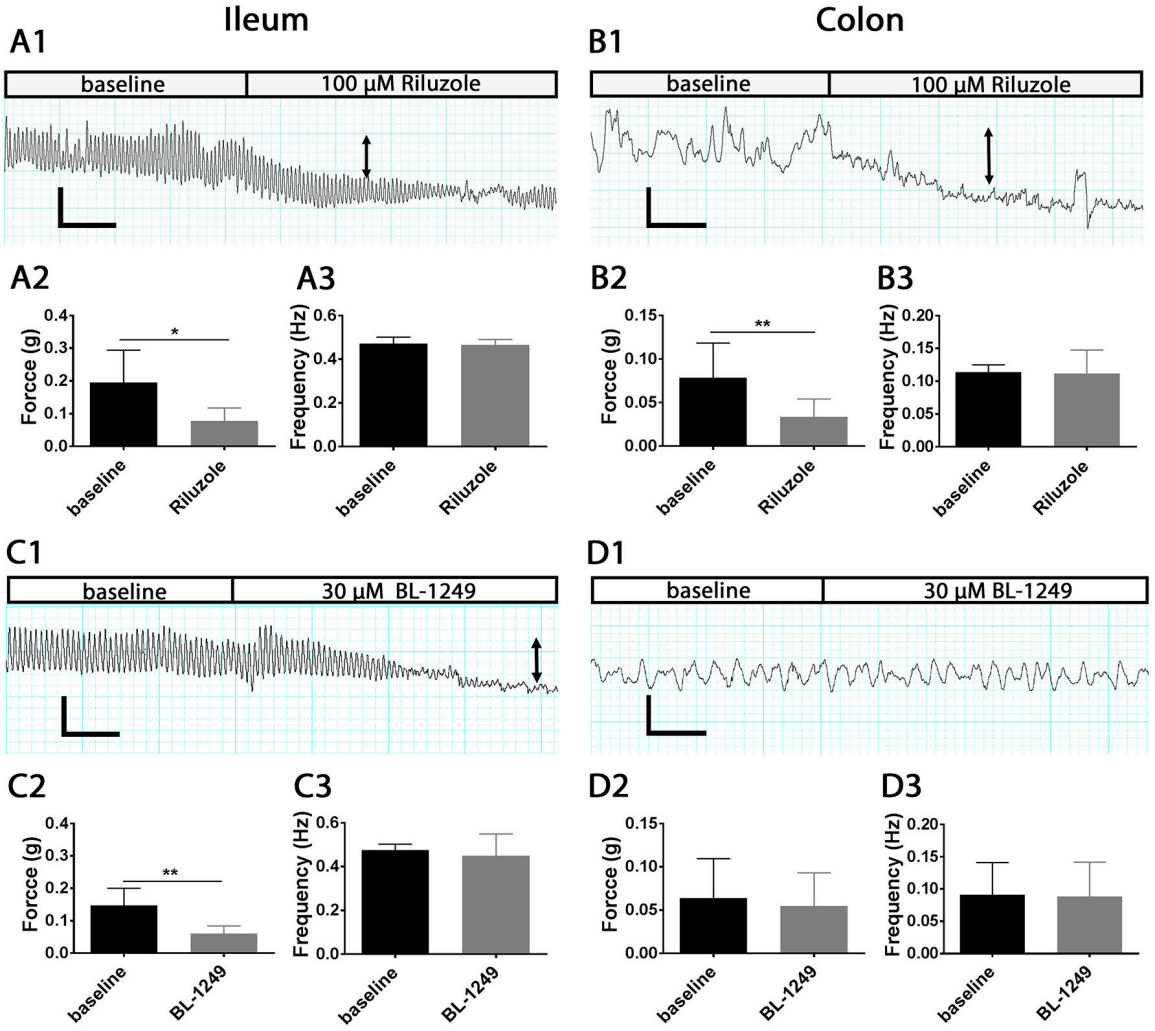
Activation of mechano-gated K_2P_ channels by Riluzole and BL-1249 reduces the force but not frequency of spontaneous contractions in mouse ileum and colon. Spontaneous contractions were recorded in the presence and absence of 100 μM Riluzole **(A,B)** and 30 μM BL-1249 **(C,D)** in ileum (*left*) and colon (*right*) as indicated. *Upper panels*, representative traces show the effects of 100 μM Riluzole on the spontaneous contractile responses obtained from mouse ileum **(A1)** and colon **(B1)**. Arrows signify reduction in basal tone. Mean data for force of contraction and frequency is displayed in chart form below the corresponding traces for ileum **(A2,A3)** and for colon **(B2,B3)**. *Lower panels*, representative traces show the effects of 30 μM BL-1249 on the spontaneous contractile responses obtained from mouse ileum **(C1)** and colon **(D1)**. Arrows signify reduction in basal tone. Mean data for force of contraction and frequency is displayed in chart form below the corresponding traces for ileum **(C2,C3)** and for colon **(D2,D3)**. For traces, scale bars represent 0.2 g (vertical) and 30 s (horizontal) for **(A1,C1)**, and 0.1 g (vertical) and 30 s (horizontal) for **(B1,D1)**. For bar charts, error bars represent standard deviation (SD); *n* = 6 animals. ^*^P < 0.05, ^**^P < 0.01, paired *t*-test.

To investigate whether the activation of mechano-gated K_2P_ channels influences physiological intestinal contractions, we applied the antidepressant drug Riluzole and the cyclooxygenase (COX) inhibitor BL-1249 to isolated mouse ileum and colon segments using the organ bath system and measured changes in the force and frequency of spontaneous contractions. Both Riluzole and BL-1249 have been shown to be potent activators of the mechano-gated K_2P_ channels TREK-1, TREK-2, and TRAAK (Duprat et al., 2000; Veale et al., 2014). Riluzole (100 μM), significantly decreased the force of spontaneous contractions in ileum (0.19 ± 0.04 g vs. 0.07 ± 0.01 g, *n* = 6, *p* < 0.05, Student's paired *t*-test, Figure 6A2) and in colon (0.07 ± 0.01 g vs. 0.03 ± 0.01 g, *n* = 6, *p* < 0.01, Student's paired *t*-test, Figure 6B2) and produced a noticeable reduction in basal tone as illustrated in the exemplar traces shown in Figures 6A1,B1. Likewise, addition of BL-1249 (30 μM) significantly reduced the force of spontaneous contractions in ileum (0.14 ± 0.02 g vs. 0.06 ± 0.01 g, *n* = 6, *p* < 0.01, Student's paired *t*-test, Figure 6C2) but not colon (0.06 ± 0.02 g vs. 0.05± 0.02 g, *n* = 6, *p* = 0.09, Student's paired *t*-test, Figure 6D2) as shown in Figures 6C1,D1 respectively. Neither Riluzole nor BL-1249 had any significant effect of the frequency of spontaneous contractions in ileum or colon (Figures 6A3,B3,C3,D3).

### Pharmacological activation of TREK family channels induces relaxation in carbachol pre-contracted ileum and colon

Given the clear physiological effects of pharmacological activation of mechano-gated K_2P_ channels on ileum and colon contractile activity, we decided to probe the impact of K_2P_ channel activation in pre-contracted tissues, employing the organ bath system to directly monitor changes in both ileum and colon muscle tension during application of a range of specific mechano-gated K_2P_ activators including the fenamate flufenamic acid (FFA), the lipoxygenase inhibitor cinnamyl 1-3,4-dihydroxy-alpha-cyanocinnamate (CDC) (Danthi et al., 2004; Veale et al., 2014) and the previously utilized compounds Riluzole and BL-1249.

**Figure 7:**
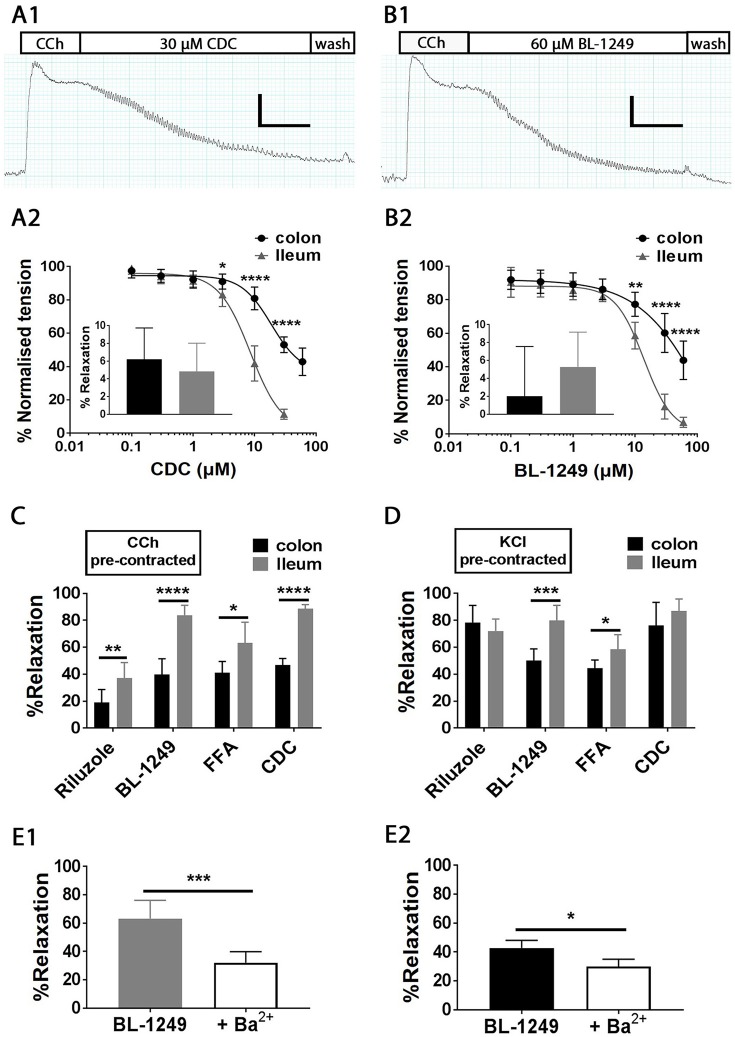
Pharmacological activation mechano-gated K_2P_ channels relaxes CCh and KCl pre-contracted mouse ileum and colon tissues. Top, representative traces showing the time course of the addition of mechano-gated K_2P_ channel activators 30 μM CDC **(A1)** and 60 μM BL-1249 **(B1)** to mouse ileum, pre-contracted with 10 μM carbachol (CCh). Scale bars represent 0.4 g (vertical), and 1 min (horizontal). Mean concentration-response relationships in CCh pre-contracted ileum (black circles) and colon (gray triangles) for Cinnamyl 1-3,4-dihydroxy-alpha-cyanocinnamate (CDC) **(A2)** and the fenamate BL-1249 **(B2)** are shown below the corresponding traces. All data was fitted with non-linear regression giving predicted IC_50_'s of CDC (8.5 ± 1.0 μM in ileum vs. 18.6 ± 3.5 μM in colon); BL-1249 (13.7 ± 1.4 μM in ileum vs. 60.8 ± 4.2 μM in colon). **(A2)** *inset*, mean normalized relaxation in CCh pre-contracted ileum (black bar) and colon (gray bar) to Zileuton (10 μM). **(B2)** *inset*, mean normalized relaxation in CCh pre-contracted ileum (black bar), and colon (gray bar) to aspirin (100 μM). **(C,D)** mean normalized relaxation of CCh **(C)** and KCl **(D)** pre-contracted ileum (black bars) and colon (gray bars) by Riluzole (100 μM), BL-1249 (30 μM), fluflenamic acid (FFA) (100 μM) and CDC (30 μM). Error bars represent standard deviation (*SD*). *Lower panels*, **(E1)** mean normalized relaxation in ileum to 10 μM BL-1249 in the absence (gray bar) and presence (white bar) of 1 mM barium chloride (Ba^2+^), **(E2)** mean normalized relaxation in colon to 30 μM BL-1249 in the absence (black bar) and presence (white bar) of 1 mM barium chloride (Ba^2+^). All data represents *n* = 6 animals. ^*^P < 0.05, ^**^P < 0.01, ^***^P < 0.001, ^****^P < 0.0001, two-way ANOVA followed by the Bonferroni *post-hoc* test **(A2,B2)**, unpaired *t*-test **(C,D)**, paired *t*-test **(E1,E2)**.

Isolated ileum and colon segments were pre-contracted with the parasympathomimetic drug Carbachol (CCh, 10 μM) and the generated tension allowed to reach steady state before the addition of K_2P_ activators (Figures 7A1,B1). Changes in tension induced by addition of activators were normalized to steady-state pre-contracted tension in each individual experiment and are reported as percent. On average 10 μM CCh generated a tension of 1.2 ± 0.03 g in ileum and 1.3 ± 0.05 g in colon (both *n* = 100). Bath exposure to the caffeic acid derivative and lipoxygenase inhibitor CDC produced a relaxation in ileum, the magnitude of which was concentration dependent (IC_50_ = 8.5 ± 1.0 μM, Figure 7A2), resulting in a 88.5 ± 1.2% relaxation at 30 μM (illustrated in the exemplar trace in Figure 7A1). Relaxation to CDC in colon was also concentration dependent (IC_50_ = 18.6 ± 3.5 μM, Figure 7A2), however the relaxations at all effective concentrations were significantly smaller than those observed in ileum, only reaching a maximally observed relaxation of 57.1 ± 3.4% at 60 μM CDC (Figure 7A2). We suggest the relaxation induced by CDC did not occur through inhibition of lipoxygenase, as application of an alternative lipoxygenase inhibitor, Zileuton (10 μM), did not induce a significant change in tension in either CCh pre-contracted ileum (4.8 ± 1.3%, *n* = 6, *p* = 0.70, Student's paired *t*-test) or colon (6.2 ± 1.4%, *n* = 6, *p* = 0.058, Student's paired *t* test) as shown in Figure 7A2 inset.

Similar to CDC, the TREK family activator and COX inhibitor BL-1249 induced a concentration dependent decrease in muscle tension in both CCh pre-contracted ileum and colon (Figure 7B2) with a predicted IC_50_ of 13.7 ± 1.4 μM (ileum, *n* = 6) and 60.8 ± 4.2 μM (colon, *n* = 6). Although relaxation induced by the highest concentration of BL-1249 used (60 μM) was not maximal, it differed significantly between ileum and colon tissues (93.1 ± 1.2% vs. 56.1 ± 4.2% respectively, *n* = 6, *p* < 0.0001, two-way ANOVA followed by a Bonferroni multiple comparisons test). The activity of BL-1249 as a K_2P_ activator was likely direct and not through accumulation of arachidonic acid induced by inhibition of COX enzymes, as application of the broad spectrum COX-inhibitor aspirin (100 μM) did not cause a significant change in tension in either CCh pre-contracted ileum (5.3 ± 1.6%, *n* = 6, *p* = 0.88, Student's paired *t*-test) or colon (2.0 ± 2.3%, *n* = 6, *p* = 0.95, Student's paired *t*-test) as shown in Figure 7B2 inset.

Single concentration additions of the related fenamate, flufenamic acid (FFA), and the antidepressant drug Riluzole also induced marked relaxations in CCh pre-contracted ileum and colon tissues, although to a lesser degree than those observed with BL-1249 and CDC [Figure 7C; FFA, 63.3 ± 6.2% (ileum) and 41.1 ± 3.4% (colon); Riluzole, 37.1 ± 4.7% (ileum) and 18.9 ± 4.0% (colon)]. Consistent with our data obtained with CDC and BL-1249, the observed relaxations to FFA and Riluzole were significantly greater in ileum vs. colon (Figure 7C, *n* = 6, *p* < 0.05, Student's unpaired *t*-test).

### Relaxation induced by TREK-1 activator BL-1249 can be attenuated by barium

There is currently a lack of well tested, specific TREK-1 inhibitors, however the non-selective potassium channel pore blocker barium chloride (Ba^2+^) has been shown to effectively inhibit TREK-1 channels with an IC_50_ of ~1 mM (Ma et al., 2011). We therefore tested the ability of the TREK-1 activator BL-1249 to relax CCh pre-contracted ileum and colon in the presence of 1 mM Ba^2+^. As would be predicted, addition of 1 mM Ba^2+^ significantly attenuated the relaxatory response to BL-1249 in both ileum (Figure 7E1) and colon (Figure 7E2). In ileum, 10 μM BL-1249 induced a mean normalized relaxation of 63.2 ± 5.3% which was reduced to 32.1 ± 3.1% in the presence of Ba^2+^ (*p* = 0.0003, Student's paired *t*-test). A similar effect was observed in colon were 30 μM BL-1249 induced a mean normalized relaxation of 42.8 ± 2.1% compared to 29.9 ± 2.4% in the presence of 1 mM Ba^2+^ (*p* = 0.0143, Student's paired *t*-test). Furthermore, under basal conditions in the absence of pre-contraction, 10 mM Ba^2+^ was able to induce significant contractile responses in both ileum (1.1 ± 0.1 g, *n* = 6) and colon (0.5 ± 0.1 g, *n* = 6).

### Relaxation by mechano-gated K_2P_ activators occurs via smooth muscle TREK-1 channels

Data from these functional studies demonstrate a clear contribution of TREK-like channels to GI tract smooth muscle contractility, however it is uncertain whether the relaxations observed with the K_2P_ activators occurred at the level of the smooth muscle (via TREK-1) or through modulation of neuronal input (via TREK-2/TRAAK). To clarify this, we performed drug additions (1) in tissues pre-contracted with potassium chloride (60 mM KCl) and (2) in tissues pre-contracted with CCh in the presence of tetrodotoxin (TTX), a voltage-gated sodium channel blocker, which in this preparation will block neuronal action potentials and prevents ENS activity (Seifi et al., 2014).

Isolated ileum and colon segments were pre-contracted with 60 mM KCl and the generated tension allowed to reach steady state before the addition of K_2P_ activators. Changes in tension induced by addition of TREK-1 activators were normalized to steady-state pre-contracted tension in each individual experiment and are reported as percent. On average 60 mM KCl generated a tension of 0.6 ± 0.04 g in ileum and 0.8 ± 0.05 g in colon (both *n* = 50). All mechano-gated K_2P_ channel activators (Riluzole, BL-1249, FFA, and CDC) induced robust relaxations of KCl pre-contracted ileum and colon tissues (Figure 7D) with BL-1249 and FFA producing significantly greater magnitudes of relaxation in ileum compared to colon (*n* = 6, *p* = <0.05, Student's unpaired *t*-test). In ileum, the extent of the relaxation induced by BL-1249, FFA, and CDC was independent of the manner of pre-contraction, however Riluzole produced a significantly greater relaxation when the ileum was pre-contracted with KCl compared to CCh (72.1 ± 3.6 vs. 37.1 ± 3.9%, *n* = 6, *p* = <0.0001, Student's unpaired *t*-test). This phenomenon was similarly observed in colon with Riluzole (78.3 ± 5.2%, KCl vs. 18.9 ± 4.4%, CCh, *n* = 6, *p* < 0.0001, Student's unpaired *t*-test) and CDC (76.2 ± 6.9% in KCl vs. 57.1 ± 3.3% in CCh, *n* = 6, *p* = <0.01, Student's unpaired *t*-test).

These data are consistent with a direct smooth muscle effect. To reinforce this hypothesis we assessed the effects of a K_2P_ activator in the presence of tetrodotoxin (TTX). TTX is a voltage-gated sodium channel blocker, which in this preparation will block neuronal action potentials and therefore prevent ENS activity (Seifi et al., 2014). The relaxation of CCh pre-contracted tissues induced by BL-1249 (60 μM) persisted in the presence of TTX (1 μM); in ileum, 91.1 ± 2.0% (without TTX) vs. 98.2 ± 3.7% (with TTX), *n* = 6, *p* = 0.038, Student's paired *t*-test; in colon, 63.6 ± 2.9% (without TTX) vs. 64.8 ± 5.0% (with TTX), *n* = 6, *p* = 0.74, Student's paired *t*-test (data not illustrated).

Overall, these data suggest that the relaxation induced by activation of mechano-gated K_2P_ channels is independent of enteric neuron input and therefore likely occurs at the level of the smooth muscle via activation of TREK-1 channels.

## Discussion

Our understanding of the expression profiles and functional roles of K_2P_ channels, particularly in smooth muscle tissue of the gastrointestinal tract is rather limited. This is largely due to a paucity of K_2P_ channel specific pharmacological tools. However, of the K_2P_ channels described to date, the mechano-gated TREK subfamily of K_2P_ channels are appearing as important regulators of smooth muscle contractility (Parelkar et al., 2010; Lembrechts et al., 2011; Monaghan et al., 2011; Lei et al., 2014) and importantly, details of several activators and inhibitors for this subfamily are emerging (Vivier et al., 2016). Previous studies have indicated that TREK-like channels underlie a stretch-dependant conductance in colonic smooth muscle cells, based on the biophysical phenotype and similarity to cloned murine TREK-1 channels, including mechano-sensitivity, insensitivity to classical potassium channel blockers, and regulation by nitrergic stimulation (Koh and Sanders, 2001; Koh et al., 2001; Park et al., 2005). However, direct pharmacological support for this hypothesis is sought. In this study we show TREK-1, TREK-2, and TRAAK channels are differentially expressed in murine ileum and colon, with TREK-1 channels displaying a primarily smooth muscle cell expression with TREK-2 and TRAAK limited to enteric neurons. Furthermore, we present clear pharmacological evidence for a functional role for TREK-1 in determining gastrointestinal contractility.

Evidence for the expression of K_2P_ channels in the gastrointestinal system is poor, with transcript profiles restricted to the TASK and TREK subfamilies. In this study we performed the first, whole family profiling of K_2P_ channel mRNA in the gastrointestinal tract. Our data showed robust transcript expression for multiple K_2P_ channels including TWIK-1, TASK-1, TASK-2, and TREK-1. The profiles of K_2P_ channel mRNA was comparable across ileum and colon, at least at the whole tissue level. A caveat to our transcript profiling was that mRNA levels were assessed in whole tissues and could not differentiate between the proportions within different cell types present in the GI tract (smooth muscle, neuron, glia, epithelia). Hence, the low level expression of some K_2P_ channel transcripts including TREK-2 and TRAAK likely represented discrete, cell specific expression profiles. Our data echoed previous RT-PCR studies, where TREK-1 (KCNK2), TASK-1 (KCNK3), TASK-2 (KCNK5), and TASK-3 (KCNK9) mRNA have been detected in colonic muscle tissue (Koh et al., 2001; Cho et al., 2005; Kubota et al., 2015). However, a novel finding from our qPCR was the high expression of TWIK-1 (KCNK1) and TASK-2 (KCNK5) mRNA in both ileum and colon. Detailed immunohistochemical analysis for these proteins demonstrated a wide distribution pattern across smooth muscle and neuronal cell types in ileum and colon and may be suggestive of important roles in gastrointestinal physiology. Indeed Cho et al. (2005) previously highlighted TASK-2 expression in murine colon and a TASK-2-like conductance as a potential contributor to resting membrane potential, although the pharmacological tools used to assess this (lidocaine) were used at concentrations too high to be TASK-2 specific. Along with a current lack of pharmacological tools for TWIK-1, the physiological importance of these two channels in the gastrointestinal system remains to be conclusively determined.

However, a physiological role of a mechano-gated K_2P_ channel has been proposed, and biophysical characteristics and transcript expression profile point toward TREK-1 as the molecular correlate of the SDK conductance in colonic myocytes (Koh and Sanders, 2001), although direct protein localization and conclusive pharmacological evidence was needed. Immunohistochemical analysis of all three mechano-gated K_2P_ channel proteins in the murine GI tract revealed a distinct expression pattern of these channels. We found evidence for TREK-1 channel expression in longitudinal and circular smooth muscle tissues, but not in nitrenergic enteric neurons of the myenteric and submucosal plexuses, whereas TREK-2 and TRAAK channel proteins were only observed in enteric neurons and were not detected in smooth muscle layers. This distinctive expression pattern is analogous to previous observations in airway (Lembrechts et al., 2011) and bladder (Lei et al., 2014) and contrasts their expression in the central nervous system where they show overlapping expression in a variety of neurons where they are thought to form functional heterodimers (Blin et al., 2016). That TREK-1 is expressed in smooth muscle and not neurons in the gut suggests a specific role for this channel in gastrointestinal physiology.

In support of our immunohistochemical findings we sought further pharmacological evidence for mechano-gated K_2P_ functional expression in murine ileum and colon using an organ bath preparation. Previously, a proposed TREK inhibitor, L-methionine, was used to suggest the functional presence of TREK channels in colonic myocytes (Park et al., 2005). However, it has since been argued that the effects of L-methionine in native tissue do not involve TREK-1 (Gil et al., 2012). Indeed, in the same study, the authors utilized a TREK-1 blocker, spadin, and observed no effect on colonic membrane potential or spontaneous motility. Given the lack of clarity and deficiency of alternative specific TREK channel inhibitors, we therefore assessed the repolarizing power of established mechano-gated K_2P_ channel activators in pre-contracted tissues and on spontaneous activity to assay the contribution of TREK channels to intestinal contractility. The approach yielded strong evidence for a functional role of smooth muscle TREK-1 in murine gastrointestinal tract, both under basal conditions and during pre-contraction.

In order to provide evidence that the relaxatory effect of the activators originated at the level of the smooth muscle we pre-contracted with potassium chloride. In the case of Riluzole, the relaxatory effect was stronger than that previously observed with CCh pre-contraction. This differential effect could be explained by activation of enteric neurons which would counteract the relaxatory response at the smooth muscle. This would also provide an explanation for the enhanced relaxation to BL-1249 observed during TTX blockade. Alternatively, the mechanism of pre-contraction could also be influential. Carbachol, a parasympathomimetic, activates metabotropic muscarinic receptors inducing contraction via: (1) transmembrane calcium influx through TRPC channels, (2) subsequent transmembrane calcium influx via VDCC, and (3) SR calcium release (Ambudkar, 2009). When using KCl as the contractile stimulus, only transmembrane calcium influx via VDCC occurs and calcium influx through TRPC channels and SR calcium release would not occur. Thus, the use of TREK-1 activators would be expected to modulate the membrane potential driven calcium influx through VDCC (receptor independent) and therefore might appear less efficacious in CCh pre-contracted tissues.

Available data suggests all utilized compounds are potent activators of mechano-gated TREK channels. Riluzole has been shown to directly activate TREK-1, TREK-2, and TRAAK channels (Duprat et al., 2000), and CDC to activate TREK-1 (Danthi et al., 2004). Fenamates, non-steroid anti-inflammatory drugs, including BL-1249 and FFA (flufenamic acid) have been revealed to directly and reversibly activate TREK-1, TREK-2, and TRAAK channels (Takahira et al., 2005; Veale et al., 2014) but have also been reported to activate a number of other potassium channels (for review see Guinamard et al., 2013). All four compounds produced comparable effects on pre-contracted ileum and colon tissues and have previously been used to describe functions of mechano-gated K_2P_ channels in native tissues including sympathetic neurons (Cadaveira-Mosquera et al., 2011) dorsal root ganglion cells (Han et al., 2016), the blood brain barrier (Bittner et al., 2013), bladder (Tertyshnikova et al., 2005), and adrenocortex (Enyeart et al., 2002; Danthi et al., 2004). However, in order to further reinforce the suggestion that the observed relaxation occurs via activation of TREK-1 channels, we investigated the impact of a known, but not specific, TREK-1 inhibitor, barium, on the ability of the TREK-1 activator BL-1249 to induce relaxation in both ileum and colon. Barium was chosen due to a lack of suitable alternative TREK-1 blockers. Spadin is a proposed TREK-1 inhibitor (Mazella et al., 2010), however, mechanistically it is an antagonist and not a classical blocker, and does not block TREK-1 currents under basal conditions but rather prevents activation (Moha Ou Maati et al., 2012). The specific molecular mechanism of action of spadin has not been described and its ability to prevent TREK-1 activation by Riluzole, FFA, CDC, and BL-1249 has not yet been established or confirmed. Fluoxetine is another potent blocker of TREK-1 (Kennard et al., 2005), however its activity as a serotonin (5-hyroxytryptamine) reuptake inhibitor would modulate contractile activity in the gastrointestinal tract through mechanisms independent of TREK-1 and therefore precluded its use. Although not specific, the IC_50_ for TREK-1 block by barium is ~1 mM (Ma et al., 2011). Pre-exposure of ileum and colon tissues to 1 mM barium attenuated the relaxatory response to the TREK-1 activator, BL-1249, by 50 and ~30% respectively, in line with what might be predicted. This provides further support for the suggestion that BL-1249 likely relaxes the gastrointestinal smooth muscle via TREK-1 activation.

Taken together these data provide the only pharmacological evidence to date for a functional role for TREK-1 channels in intestinal contractility, and coupled with clear molecular and protein expression profiles, could have potential therapeutic implications for treating motility disorders arising from hyper-contractility, such as irritable bowel syndromes.

## Author contributions

Conceived and designed the experiments: RM and AL; completion of experiments: RM, MS, and MP; analysis and interpretation of data: RM, MS, MP, AL, and JS; wrote the draft manuscript: RM and AL. Revised the manuscript: JS, JB, MP, and MS. All authors reviewed and approved the manuscript.

### Conflict of interest statement

The authors declare that the research was conducted in the absence of any commercial or financial relationships that could be construed as a potential conflict of interest.

## Abbreviations

4-AP: 4-aminopyridine
BKCa: Large conductance calcium-activated potassium channel
BL-1249: (5,6,7,8-tetrahydro-naphthalen-1-yl)-[2-(1H-tetrazol-5-yl)-phenyl]-amine
CCh: Carbachol
CDC: Cinnamyl-3,4-dihydroxy-α-cyanocinnamate
DIC: Differential interference contrast
FFA: Flufenamic acid
hERG: Human *ether-a-go-go* potassium channel
IBS: Irritable Bowel Syndrome
K_1P_: Single pore-domain potassium
K_2P_: Two pore-domain potassium
NOS: Nitric oxide synthase
qPCR: Quantitative Polymerase Chain Reaction
SDK: Stretch-dependent potassium channel
TBS-Tx: Tris Buffer Saline containing Triton X-100
TEA: Tetraethylammonium
TTX: Tetrodotoxin
VDCC: Voltage-dependent calcium channel.
