# Supplementary material for: TREK-1 Channel Expression in Smooth Muscle as a Target for Regulating Murine Intestinal Contractility: Therapeutic Implications for Motility Disorders
Source: Front Physiol. 2018 Mar 6;9:157. doi: 10.3389/fphys.2018.00157 (PMC5845753; doi:10.3389/fphys.2018.00157)
Supplement: Supplementary file 1 [file Table1.DOCX]

| **Target** | **Forward Primer 5’ – 3’** | **Reverse Primer 3’ – 5’** | **Accession Number** |
| --- | --- | --- | --- |
| KCNK1 (TWIK-1) | GAAGTTCAGGAAGATGTTCTACG | GTGACGGAGGAGAAGGACAG | NM_008430 |
| KCNK2 (TREK-1) | CGAAGGAAGAGGTGGGAGAG | TGGCACGCTGGAACTTGT | NM_00115985 |
| KCNK3 (TASK-1) | GACTGCCCTTGGAATCTAAAACA | CAACCTATGGACGAGTGAATGAG | NM_010608 |
| KCNK4 (TRAAK) | GCTCTTCCCTGGCTGGTTC | GGTTTTGAGGCACAGTTGTGA | NM_008431 |
| KCNK5 (TASK-2) | GGTGACAGAAGAATGGAACTACA | GTATCGGTATAGGGCGTGGTA | NM_021542 |
| KCNK6 (TWIK-2) | TGTTTTGTTTTGTTCTTGTTGTTTTTC | CTAATACAGAGGCTAAAGTGGAGTC | NM_00103352 |
| KCNK7  (K2P 7.1) | CCTCAGTATCAGAACATCAGAACAG | TATTCATTCCCATAACCCCTTCCT | NM_010609 |
| KCNK9 (TASK-3) | GTTCCTTCTACTTCGCCATCAC | GCCTTGCCAGCATCAGTTC | NM_00103387 |
| KCNK10 (TREK-2) | AAAGAAGCAAGTGAGCCAGAC | ACAGCAGGGATGGTCACAAA | NM_029911 |
| KCNK12 (THIK-2) | TACCGCCTGGGCAACTTC | GCTTGATGAGGATGGAGATGAC | NM_199251 |
| KCNK13 (THIK-1) | AGAGGCACCGAAGACAGAGA | GGTCGTGGGTCCAGAGAGA | NM_00116442 |
| KCNK15 (TASK-5) | TCATCACTCTCACCACCATAGG | CCACCAGGTTGAGGAAGGC | NM_00103029 |
| KCNK16 (TALK-1) | CTGGCTCTGTTCCTGACCTT | CAATGGTGCTGAGGGTGATAAA | NM_029006 |
| KCNK18 (TRESK) | TCTGCTTTGTGACACTGACCA | TGTAGATGGAGAAGAAGAGGAAGAA | NM_207261 |
| HPRT | TCCTCCTCAGACCGCTTTT | AGGTATACAAAACAAATCTAGGTCAT | NM_013556 |
| RPLP0 | AGAGTCGGAGGAATCAGATGA | GAGTTTTAAGAGAAGTAAGCCTTTATT | NM_007475 |
| GAPDH |  |  | NM_008084.2 |

**Supplementary Table 1. Primer sequences for qPCR analysis.** Primers were custom designed (Primerdesign Ltd, Chandlers Ford, UK). Forward and reverse primer pair sequences are shown with corresponding GenBank^®^ accession numbers for the target genes. Specific sequence information for GAPDH is unavailable, as the geNorm reference gene assay contains commercially sensitive information. However, the context information provided for mouse GAPDH includes: species, mouse, accession number, NM_008084.2, anchor nucleotide, 793, and context sequence length, 180 bp.
