# Supplementary material for: TREK-1 Channel Expression in Smooth Muscle as a Target for Regulating Murine Intestinal Contractility: Therapeutic Implications for Motility Disorders
Source: Front Physiol. 2018 Mar 6;9:157. doi: 10.3389/fphys.2018.00157 (PMC5845753; doi:10.3389/fphys.2018.00157)
Supplement: Supplementary file 2 [file Table2.DOCX]

| **Primary antibody** | **Host** | **Source** | **Dilution** | **Specificity/Reference** | **Secondary antibody** |
| --- | --- | --- | --- | --- | --- |
| NOS | Sheep polyclonal | Millipore (AB1529) | 1:1000 | ([Cauli et al., 2004](#_ENREF_4);[Liu et al., 2008](#_ENREF_8)) | Donkey anti-sheep Alexa 488 |
| ChAT | Goat polyclonal | Millipore (AB144P) | 1:1000 | Heinze et al., 2007; Härtig et al., 2007 | Donkey anti-goat Alexa 488 |
| CR | Goat polyclonal | Swant (CG1) | 1:1000 | ([Schiffmann et al., 1999](#_ENREF_9)) | Donkey anti-goat Alexa 488 |
| α-actinin | Mouse monoclonal | Sigma-Aldrich (A5044) | 1:500 | ([Hawkins et al., 2008](#_ENREF_6)) | Donkey anti-mouse cy5 |
| TWIK-1 | Rabbit polyclonal | Alomone Labs (APC-110) | 1:1000 | ([Zhou et al., 2009](#_ENREF_12);[Wang et al., 2013](#_ENREF_10)) | Donkey anti-rabbit cy3 |
| TASK-2 | Rabbit polyclonal | Alomone Labs (APC-037) | 1:1000 | ([Cho et al., 2005](#_ENREF_5)) | Donkey anti-rabbit cy3 |
| TREK-1 | Rabbit polyclonal | Alomone Labs (APC-047) | 1:1000 | ([Banerjee et al., 2016](#_ENREF_2);[Blin et al., 2016](#_ENREF_3)) | Donkey anti-rabbit cy3 |
| TREK-2 | Rabbit polyclonal | Alomone Labs (APC-055) | 1:1000 | ([Acosta et al., 2014](#_ENREF_1);[Blin et al., 2016](#_ENREF_3)) | Donkey anti-rabbit cy3 |
| TRAAK | Rabbit polyclonal | Alomone Labs (APC-108) | 1:1000 | ([Hur et al., 2012](#_ENREF_7);[Zhang et al., 2015](#_ENREF_11)) | Donkey anti-rabbit cy3 |

**Supplementary Table 2. Details of primary and secondary antibodies used for immunohistochemistry.** The table includes the dilutions used for each antibody and references of studies were specificity of the antibodies has been confirmed. NOS, nitric oxide synthase; ChAT, choline acetyltransferase; CR, calretinin.

**References associated with Suppl Table 2.**

Acosta, C., Djouhri, L., Watkins, R., Berry, C., Bromage, K., and Lawson, S.N. (2014). TREK2 expressed selectively in IB4-binding C-fiber nociceptors hyperpolarizes their membrane potentials and limits spontaneous pain. *J Neurosci* 34**,** 1494-1509.

Banerjee, A., Ghatak, S., and Sikdar, S.K. (2016). l-Lactate mediates neuroprotection against ischaemia by increasing TREK1 channel expression in rat hippocampal astrocytes in vitro. *J Neurochem* 138**,** 265-281.

Blin, S., Ben Soussia, I., Kim, E.J., Brau, F., Kang, D., Lesage, F., and Bichet, D. (2016). Mixing and matching TREK/TRAAK subunits generate heterodimeric K2P channels with unique properties. *Proc Natl Acad Sci U S A* 113**,** 4200-4205.

Cauli, B., Tong, X.K., Rancillac, A., Serluca, N., Lambolez, B., Rossier, J., and Hamel, E. (2004). Cortical GABA interneurons in neurovascular coupling: relays for subcortical vasoactive pathways. *J Neurosci* 24**,** 8940-8949.

Cho, S.Y., Beckett, E.A., Baker, S.A., Han, I., Park, K.J., Monaghan, K., Ward, S.M., Sanders, K.M., and Koh, S.D. (2005). A pH-sensitive potassium conductance (TASK) and its function in the murine gastrointestinal tract. *J Physiol* 565**,** 243-259.

Hawkins, T.A., Haramis, A.P., Etard, C., Prodromou, C., Vaughan, C.K., Ashworth, R., Ray, S., Behra, M., Holder, N., Talbot, W.S., Pearl, L.H., Strahle, U., and Wilson, S.W. (2008). The ATPase-dependent chaperoning activity of Hsp90a regulates thick filament formation and integration during skeletal muscle myofibrillogenesis. *Development* 135**,** 1147-1156.

Hur, C.G., Kim, E.J., Cho, S.K., Cho, Y.W., Yoon, S.Y., Tak, H.M., Kim, C.W., Choe, C., Han, J., and Kang, D. (2012). K(+) efflux through two-pore domain K(+) channels is required for mouse embryonic development. *Reproduction* 143**,** 625-636.

Liu, S., Qu, M.H., Ren, W., Hu, H.Z., Gao, N., Wang, G.D., Wang, X.Y., Fei, G., Zuo, F., Xia, Y., and Wood, J.D. (2008). Differential expression of canonical (classical) transient receptor potential channels in guinea pig enteric nervous system. *J Comp Neurol* 511**,** 847-862.

Schiffmann, S.N., Cheron, G., Lohof, A., D'alcantara, P., Meyer, M., Parmentier, M., and Schurmans, S. (1999). Impaired motor coordination and Purkinje cell excitability in mice lacking calretinin. *Proc Natl Acad Sci U S A* 96**,** 5257-5262.

Wang, W., Putra, A., Schools, G.P., Ma, B., Chen, H., Kaczmarek, L.K., Barhanin, J., Lesage, F., and Zhou, M. (2013). The contribution of TWIK-1 channels to astrocyte K(+) current is limited by retention in intracellular compartments. *Front Cell Neurosci* 7**,** 246.

Zhang, J., Cao, M., Wu, X., Chen, Y., Liang, W., and Liang, Y. (2015). Enhanced expression of TWIK-related arachidonic acid-activated K+ channel in the spinal cord of detrusor overactivity rats after partial bladder outlet obstruction. *BMC Urol* 15**,** 100.

Zhou, M., Xu, G., Xie, M., Zhang, X., Schools, G.P., Ma, L., Kimelberg, H.K., and Chen, H. (2009). TWIK-1 and TREK-1 are potassium channels contributing significantly to astrocyte passive conductance in rat hippocampal slices. *J Neurosci* 29**,** 8551-8564.
