# Supplementary material for: TREK-1 Channel Expression in Smooth Muscle as a Target for Regulating Murine Intestinal Contractility: Therapeutic Implications for Motility Disorders
Source: Front Physiol. 2018 Mar 6;9:157. doi: 10.3389/fphys.2018.00157 (PMC5845753; doi:10.3389/fphys.2018.00157)
Supplement: Supplementary file 3 [file Image1.PDF]

Ma *et al.*, 2017; Supplementary Figure 1

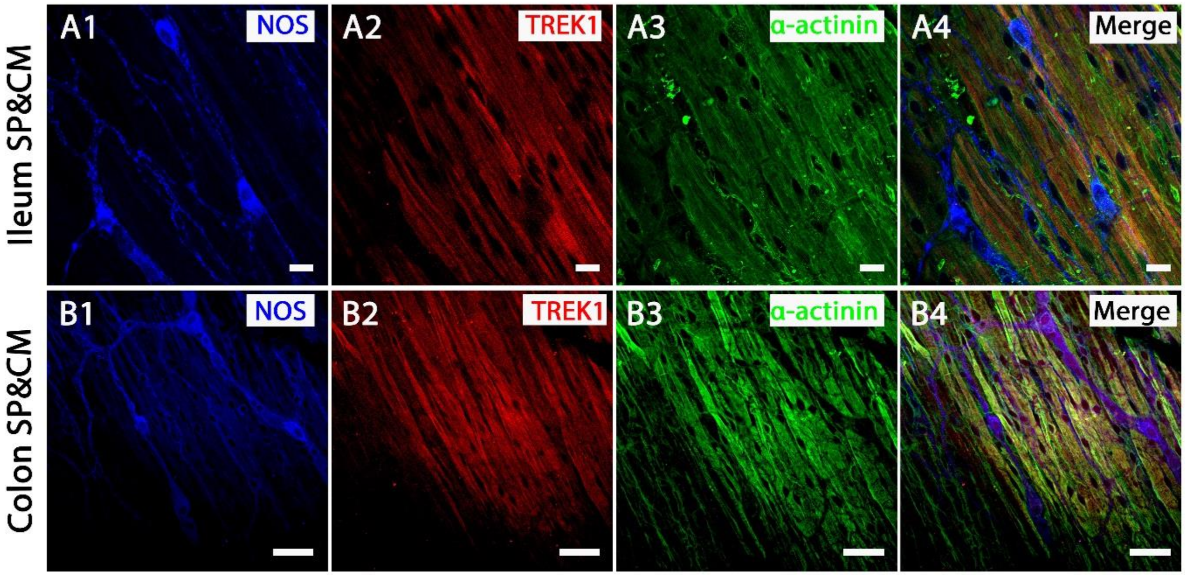

Supplementary Figure 1. TREK-1 channels are expressed in circular smooth muscle of both mouse ileum and colon. Images show the expression of NOS immunoreactive submucosal plexus neurons (*blue*, **A1** and **B1**), TREK-1 channel (*red*, **A2** and **B2**) and α-actinin immunoreactive smooth muscle cells (*green*, **A3** and **B3**) in mouse ileum and colon as indicated. **(A4)** and **(B4)** are overlays of **(A1-3) and (B1-B3)** respectively indicating smooth muscle expression of TREK-1 channels in circular smooth muscle layers of the mouse ileum and colon. Scale bar represents 20 μm **(A1-A4)** and 50 μm **(B1-B4)**.
