# Supplementary material for: TREK-1 Channel Expression in Smooth Muscle as a Target for Regulating Murine Intestinal Contractility: Therapeutic Implications for Motility Disorders
Source: Front Physiol. 2018 Mar 6;9:157. doi: 10.3389/fphys.2018.00157 (PMC5845753; doi:10.3389/fphys.2018.00157)
Supplement: Supplementary file 4 [file Image2.PDF]

Ma *et al.*, 2017; Supplementary Figure 2

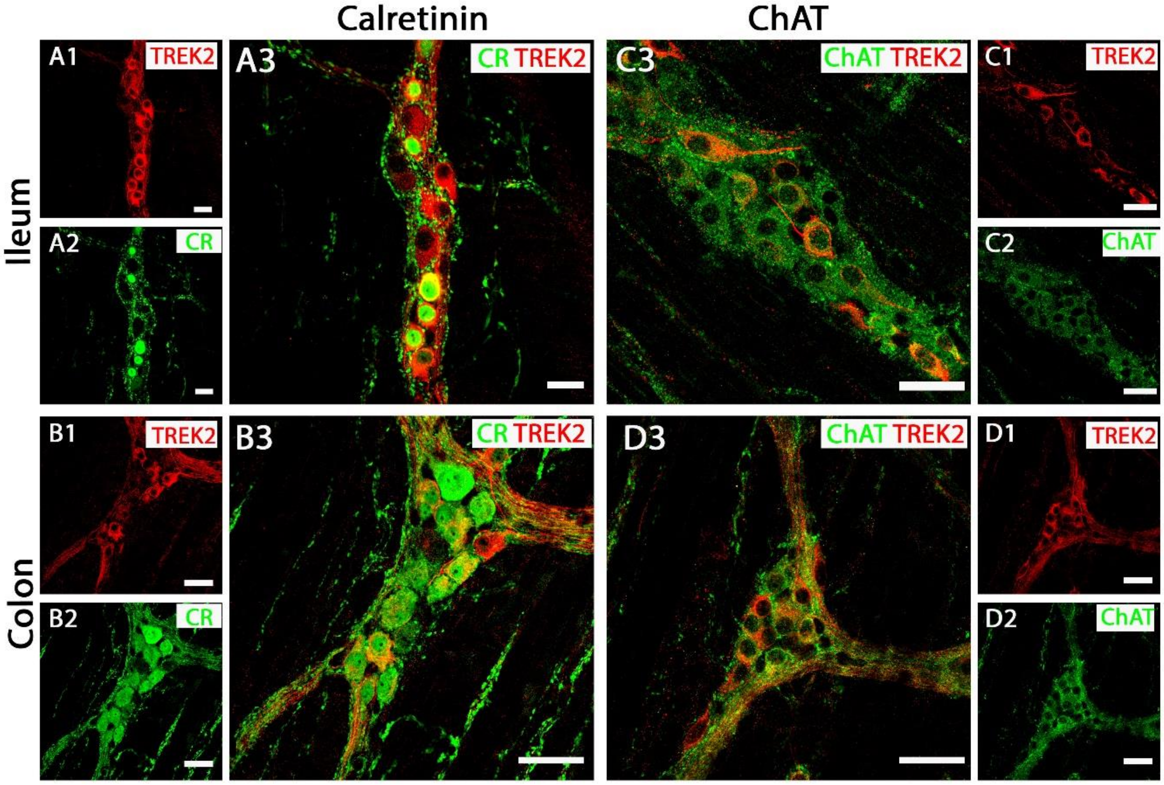

Supplementary Figure 2. TREK-2 channels are expressed in ChAT-positive and calretinin-positive myenteric plexus neurons in mouse ileum and colon. Panels **A3** and **C3** (ileum) and **B3** and **D3** (colon) show overlays of cell specific markers (green) and the TREK-2 channel marker (red). For clarity, panels **A1** and **A2** (ileum) and **B1** and **B2** (colon) show the individual images from corresponding merges in **A3** and **B3**, panels **C1** and **C2** (ileum) and **D1** and **D2** (colon) show the individual images from corresponding merges in **C3** and **D3**. Overlays shown in **A3** and **B3** reveal TREK-2 channels are expressed in calretinin-immunopositive and calretinin-immunonegative enteric neurons in mouse ileum and colon. Overlays shown in **C3** and **D3** indicate TREK-2 channels are expressed in ChAT-immunopositive excitatory neurons and ChAT-immunonegative neurons in mouse ileum and colon. Scale bars represent 20 μm **(A1-A3)**, 40 μm **(B1-B3),** 30 μm **(C1-C3)** and 40 μm **(D1-D4)**.
